# Biotransformation of Liquiritigenin into Characteristic Metabolites by the Gut Microbiota

**DOI:** 10.3390/molecules27103057

**Published:** 2022-05-10

**Authors:** Adili Keranmu, Li-Bin Pan, Jie Fu, Pei Han, Hang Yu, Zheng-Wei Zhang, Hui Xu, Xin-Yu Yang, Jia-Chun Hu, Hao-Jian Zhang, Meng-Meng Bu, Jian-Dong Jiang, Nian-Zeng Xing, Yan Wang

**Affiliations:** 1 State Key Laboratory of Molecular Oncology, Department of Urology, National Cancer Center/National Clinical Research Center for Cancer/Cancer Hospital, Chinese Academy of Medical Sciences and Peking Union Medical College, Beijing 100021, China; adili117@163.com; 2 State Key Laboratory of Bioactive Substance and Function of Natural Medicines, Institute of Materia Medica, Chinese Academy of Medical Sciences/Peking Union Medical College, Beijing 100050, China; panlibin@imm.ac.cn (L.-B.P.); fujie@imm.ac.cn (J.F.); hanpei@imm.ac.cn (P.H.); yuhang@imm.ac.cn (H.Y.); zhangzhengwei@imm.ac.cn (Z.-W.Z.); xuhui@imm.ac.cn (H.X.); xinyuy20@mails.jlu.edu.cn (X.-Y.Y.); hujiachu@imm.ac.cn (J.-C.H.); zhjzlh@126.com (H.-J.Z.); bmmzqq@163.com (M.-M.B.); 3 Department of Urology, Shanxi Province Cancer Hospital/Shanxi Hospital Affiliated to Cancer Hospital, Chinese Academy of Medical Sciences/Cancer Hospital Affiliated to Shanxi Medical University, Beijing 100006, China

**Keywords:** liquiritigenin, gut microbiota, liver microsome, metabolites

## Abstract

The bioavailability of flavonoids is generally low after oral administration. The metabolic transformation of flavonoids by the gut microbiota may be one of the main reasons for this, although these metabolites have potential pharmacological activities. Liquiritigenin is an important dihydroflavonoid compound found in *Glycyrrhiza uralensis* that has a wide range of pharmacological properties, such as antitumor, antiulcer, anti-inflammatory, and anti-AIDS effects, but its mechanism of action remains unclear. This study explored the metabolites of liquiritigenin by examining gut microbiota metabolism and hepatic metabolism in vitro. Using LC-MS/MS and LC/MS^n^-IT-TOF techniques, three possible metabolites of liquiritigenin metabolized by the gut microbiota were identified: phloretic acid (M3), resorcinol (M4), and M5. M5 is speculated to be davidigenin, which has antitumor activity. By comparing these two metabolic pathways of liquiritigenin (the gut microbiota and liver microsomes), this study revealed that there are three main metabolites of liquiritigenin generated by intestinal bacteria, which provides a theoretical basis for the study of pharmacologically active substances in vivo.

## 1. Introduction

Tumors are present in major diseases that endanger human health worldwide. There are many drugs available to treat tumor diseases in the clinic. However, clinical chemotherapy drugs have some disadvantages, such as high cost and many side effects. Therefore, there is an urgent need to find drugs with a low cost and few side effects. Traditional herbal medicines have attracted the attention of researchers in many countries as potential alternative resources to treat various diseases. Flavonoids are compounds with many biological properties, such as anti-inflammatory [1], antitumor [2], and antioxidation effects [3], that widely exist in nature. Among them, research on the anti-inflammatory and antitumor effect of flavonoids is the most extensive and in-depth. Intake of more flavonoids and flavonoid-rich foods is associated with reduced mortality from specific vascular diseases and cancers. A study from Australia using the most comprehensive flavonoid database to randomly select 1063 women over the age of 75 and followed up for more than 5 years to analyze the correlation between all-causes cancer and cardiovascular mortality found that high intake of flavonoids is associated with a reduced risk of death in older women, and it is concluded that the benefits of flavonoids may extend to the etiology of cancer and cardiovascular disease [4]. A study from Korea found that individuals with CYP1A1 variants may gain different benefits from the intake of dietary flavonoid subclasses (flavonols, flavones, flavanones, flavan-3-ols, anthocyanidins, and isoflavones) to prevent colorectal cancer [5]. In addition, the antioxidation of flavonoids is also an important biological effect. A randomized, double-blind, placebo-controlled, parallel clinical study of 92 moderately trained healthy men and women found that supplementation with citrus flavonoid extract could improve the anaerobic capacity and peak power of moderately trained individuals during high-intensity exercise [6]. Liquiritigenin, a flavonoid, is expected to be developed into an anticancer drug with low toxicity because of its antioxidant, antibacterial, antifungal, antitumor, antiproliferative, and cytotoxic activities [7,8,9,10,11].

The trillions of microorganisms in the human gut affect the normal physiology and the progression of diseases through the interaction of metabolic activities with the host [12,13]; this system is called the “invisible organ” of the human body [14]. The intestinal flora participates in various important metabolic processes in the human body, such as protein metabolism, carbohydrate metabolism, and bile acid metabolism, by providing various enzymes [15,16,17]. Certain intestinal flora can also take part in the metabolism of some oral drugs, thus changing their activity or toxicity [18]. Therefore, the gut microbiota plays an important role in the metabolic processes of the human body.

R. Kupfer et al. found that liquiritigenin metabolism produces 7,4’-dihydroxyflavone through the in vitro metabolism of human liver microsomes of liquiritigenin. However, after oral gavage and intravenous injection, they found that no metabolite was detected in most plasma samples, and only the metabolite was occasionally detected in urine and feces, suggesting that the phase II metabolism of liquiritigenin may be related to it [19]. Intestinal microorganism research has been both popular and difficult in recent years. Through the pharmacokinetics of isoliquiritigenin and its metabolites in rats, Y. K. Lee found that the low bioavailability was mainly due to liver and intestinal metabolism [20]. Liquiritigenin is a flavonoid drug extracted from the Chinese herbal medicine *Glycyrrhiza uralensis*. Its broad-spectrum biological effects may occur due to the variety of metabolites it produces in both the intestinal flora and liver. However, this hypothesis remains to be verified. Therefore, in this study, liquiritigenin was metabolized in vitro by the gut microbiota and liver microsomes, and its metabolites were analyzed by mass spectrometry to explain its possible metabolic pathways.

## 2. Results

**Figure 1:**
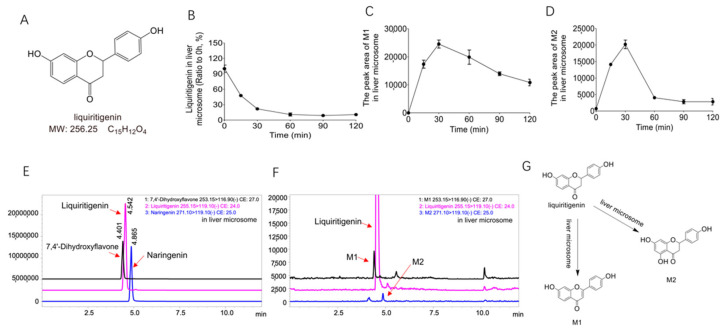
The metabolites of liquiritigenin from liver microsomes. (**A**): The chemical structure of liquiritigenin; (**B**): the relative abundance curve of liquiritigenin incubated with rat liver microsomes at different time points (0 min, 15 min, 60 min, 90 min, and 120 min); (**C**,**D**): relative abundance curves of metabolites M1 and M2 after incubation with rat liver microsomes for different lengths of time (0 min, 15 min, 60 min, 90 min, and 120 min); (**E**): extracted ion chromatograms (EICs) of the standard liquiritigenin, 7,4*’*-dihydroxyflavone, and naringenin; (**F**): EICs of liquiritigenin and its metabolites after 60 min of liver microsomal metabolism; (**G**): metabolic pathway of liquiritigenin in liver microsomes (liquiritigenin produces M1 and M2 in liver microsomes).

In this study, we aimed to explain the unique metabolic profile of liquiritigenin in the gut microbiota and compare the differences in the metabolic pathways in the gut and liver. To compare these two metabolic pathways, we wanted to explore the metabolites produced by liquiritigenin. Figure 1A shows the molecular structure of liquiritigenin. LC/MS^n^-IT-TOF and LC–MS/MS techniques were used to identify the metabolites of liquiritigenin after incubation with liver microsomes and the gut microbiota, summarize its possible cleavage pathways, and compare the metabolites obtained from the two systems to determine the metabolic characteristics of liquiritigenin in vitro.

### 2.1. Biotransformation of Liquiritigenin in Liver Microsomes

To explore the metabolism of liquiritigenin in liver microsomes, we performed in vitro metabolism experiments using a liver microsome incubation system (5 μL of Sprague–Dawley rat liver microsomes + 2 μL of liquiritigenin + 20 μL of NADPH + 173 μL of Tris/HCl). The relative abundance of liquiritigenin in the liver microsome incubation system over time is shown in Figure 1B. Figure 1B shows that liquiritigenin could be metabolized by liver microsomes, as this compound was basically metabolized within 2 h.

Interestingly, we found two metabolites (M1 and M2) in the liver microsomal culture system. Figure 1C,D show the changes metabolites M1 and M2 underwent in the liver microsomal incubation system at different time points (0 min, 15 min, 60 min, 90 min, 120 min). After comparison of the extracted ion chromatograms (EICs) (Figure 1F) of metabolites M1 and M2 and the EICs (Figure 1E) of the standard 7,4’-dihydroxyflavone and naringenin, M1 and M2 were determined to be 7,4’-dihydroxyflavone and naringenin, respectively. The parent ion *m*/*z* and product ion *m*/*z* of M1 are 253.15 and 116.90, and the retention time is 4.412 min; the parent ion *m*/*z* and product ion *m*/*z* of M2 are 271.10 and 119.10, and the retention time is 4.868 min; the parent ion *m*/*z* and product ion *m*/*z* of the standard 7,4’-dihydroxyflavone are 253.15 and 116.90, and the retention time is 4.401 min; the parent ion *m*/*z* and product ion *m*/*z* of the standard naringenin are 271.10 and 119.10, and the retention time is 4.865 min. It can also be seen that the metabolites M1 and M2 are consistent with the mass spectrometry data and retention times of the standard 7,4’-dihydroxyflavon and naringenin.

### 2.2. Biotransformation of Liquiritigenin in the Gut Microbiota

**Figure 2:**
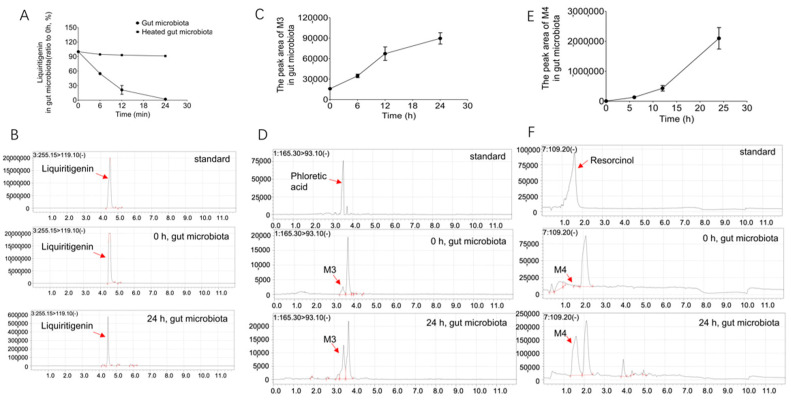
The metabolites of liquiritigenin from the gut microbiota. (**A**): The relative abundance of liquiritigenin after incubation with rat gut microbiota and heated gut microbiota for 0 h, 6 h, 12 h, and 24 h; (**B**): EIC of the standard liquiritigenin and EICs of liquiritigenin after 0 h and 24 h of incubation in the rat gut microbiota system; (**C**,**E**): time-dependent curves of metabolites M3 and M4 after incubation with rat intestinal flora for 0 h, 6 h, 12 h, and 24 h; (**D**,**F**): EICs of the standard phloretic acid and resorcinol and EICs of metabolites M3 and M4 after 0 h and 24 h of incubation in the rat intestinal flora system.

To explore whether the intestinal flora is involved in the metabolism of liquiritigenin, the colonic contents of three Sprague–Dawley (SD) rats were incubated with liquiritigenin. Secondary heating inactivated the colon content in the culture system, which was used as the negative control to eliminate the interference of environmental factors such as the culture medium. The reaction was stopped after 0 h, 6 h, 12 h, and 24 h of incubation. The changes liquiritigenin underwent in the incubation system were detected by LC/MS-8060, as shown in Figure 2A. Figure 2A shows that the relative abundance of liquiritigenin in the in vitro incubation system gradually decreased over time. In contrast, the heat-inactivated gut microbiota showed little metabolism of liquiritigenin (Figure 2A). This result suggests that the gut microbiota can metabolize liquiritigenin, shows that the decline in the liquiritigenin content is a result of cometabolism in the gut microbiota, and indicates the role of the gut microbiota in the metabolism of liquiritigenin.

Three liquiritigenin metabolites were obtained from the gut microbiota system (M3, M4 and M5) using LC/MS-8060 and LC/MS^n^-IT-TOF analyses. The changes in the relative abundance of liquiritigenin over time after 0 h, 6 h, 12 h, and 24 h of incubation with rat intestinal flora are shown in Figure 2A. It can be seen from the figure that liquiritigenin was basically metabolized within 24 h. Figure 2B shows the EICs of liquiritigenin at 0 h and 24 h and the EICs of the standard liquiritigenin. We found that liquiritigenin produced two metabolites, M3 and M4, under the actions of the intestinal flora. Figure 2C,E shows the time-dependent curves of metabolites M3 and M4 after incubation with the rat intestinal flora for 0 h, 6 h, 12 h, and 24 h. It can be seen from the figure that the abundances of M3 and M4 gradually increased with time in this system. After comparison of the EICs of metabolites M3 and M4 at 0 h and 24 h and the EICs of the standard phloretic acid and resorcinol (as shown in Figure 2D,F), M3 and M4 were determined to be phloretic acid and resorcinol, respectively. The parent ion *m*/*z* and product ion *m*/*z* of M3 are 165.30 and 93.10, and the retention time is 3.452 min; the parent ion *m*/*z* of M4 is 109.20 (no product ion), and the retention time is 1.587 min; the parent ion *m*/*z* and product ion *m*/*z* of the standard phloretic acid are 165.30 and 93.10, and the retention time is 3.462 min; the parent ion *m*/*z* of the standard resorcinol is 109.20 (no product ion), and the retention time is 1.606 min. It can also be seen that the metabolites M3 and M4 are consistent with the mass spectrometry data and retention times of the standard phloretic acid and resorcinol.

**Figure 3:**
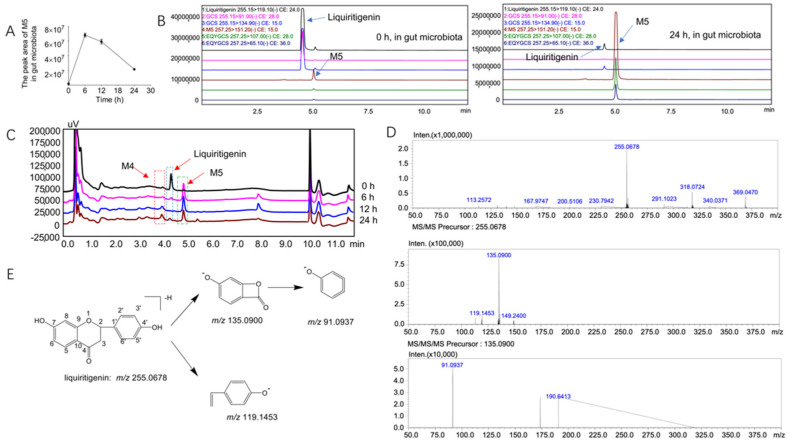
The metabolites of M5 in the gut microbiota and the mass spectrometric cleavage pathway of liquiritigenin. (**A**): Time-dependent curve of M5 after incubation with rat intestinal flora for 0 h, 6 h, 12 h and 24 h; (**B**): EICs of parent drug liquiritigenin and metabolite M5 after 0 h and 24 h of incubation in the rat intestinal flora system; (**C**): liquid EICs of liquiritigenin and metabolites M4 and M5 after 0 h, 6 h, 12 h, and 24 h of incubation; (**D**): MSn data of liquiritigenin; (**E**): possible cleavage of liquiritigenin.

**Figure 4:**
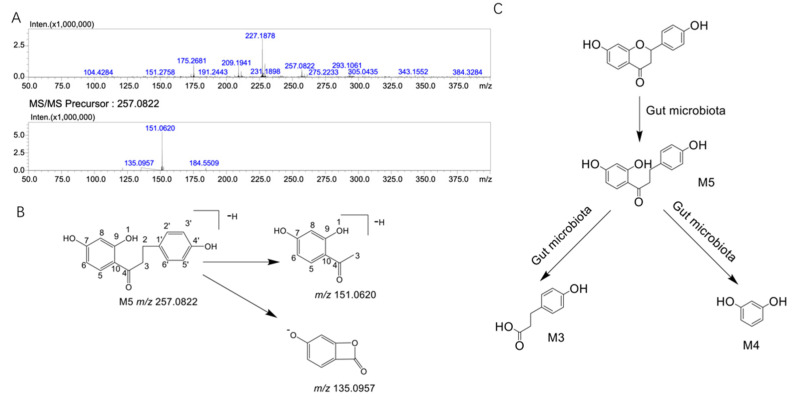
MS^n^ data of the liquiritigenin metabolite M5 and the metabolic pathway of liquiritigenin in the gut microbiota. (**A**): MS^n^ data of liquiritigenin metabolite M5; (**B**): possible cleavage of liquiritigenin metabolite M5; (**C**): metabolic pathway of liquiritigenin in the gut microbiota system (liquiritigenin is metabolized in the gut microbiota to produce M3, M4, and M5).

We found a new metabolite of liquiritigenin, M5, in the intestinal flora system. The time-dependent changes in metabolite M5 in the rat gut microbiota at 0 h, 6 h, 12 h, and 24 h are shown in Figure 3A. It can be seen from the figure that M5 first increased and then decreased over time; therefore, we speculated that M5 was further metabolized into other substances. Further LC/MS-8060 (Figure 3B) and LC/MS^n^-IT-TOF (Figure 3C) analyses showed that M5 was indeed produced (Figure 4A shows the MSn data of metabolite M5 in the gut microbiota system, whereas the MSn information of liquiritigenin and its metabolites is shown in Table 1). Based on the assumed cleavage pathway, M5 may be further broken down into M3 and M4 (Figure 4C).

### 2.3. Structural Analysis of the Unknown Metabolite M5

Mass spectrometric analysis of liquiritigenin: the [M-H]^-^ peak of liquiritigenin was found at 255.0678, and the fragments with *m*/*z* values of 135.0900 and 119.1453 were obtained from the secondary fragments (the MS^n^ data of liquiritigenin are shown in Figure 3D). It was speculated that M5 was formed by the cleavage of the C–O bonds at positions 1 and 2 and the C–C bonds at positions 3 and 4 of the parent compound. The secondary fragment at *m*/*z* 135.0900 produced a fragment at *m*/*z* 91.0937 after the loss of 44 Da (the proposed cleavage pathway of liquiritigenin is shown in Figure 3E).

Structural analysis of M5: The [M-H]^-^ peak of M5 was found at 257.0822, which corresponds to the addition of two hydrogen to the liquiritigenin structure. According to the literature, dihydroflavonoids undergo ring opening at positions 1 and 2 under the action of chalcone isomerase (CHI) and phloretin hydrolase (PHY) and then produce metabolites through hydrogenation and reduction reactions [21]. It was therefore speculated that the structure of M5 may be dihydroisoflavone. The *m*/*z* of the secondary fragment of M5 was found at 151.0620, showing a loss of 106 Da from the parent ion, which may be due to the loss of p-methylphenol (the MSn data of metabolite M5 are shown in Figure 4A). The possible cleavage pathway of M5 is shown in Figure 4B. According to the fragmentation of M5 and the metabolic trend of M5 in the intestinal flora, we infer that M5 may be further decomposed into M3 and M4.

In conclusion, through LC/MS-8060 and LC/MS^n^-IT-TOF analyses, liquiritigenin produces three metabolites, M3, M4, and M5, after metabolism in the intestinal flora, and the possible metabolic pathways are shown in Figure 4C. Table 2 shows the accurate mass measurements of molecular and fragment ions of liquiritigenin and its metabolites in the intestinal microflora metabolic system and liver microparticle incubation system.

## 3. Discussion

The bioavailability of flavonoids is generally low after oral administration. The metabolic transformation of flavonoids by the gut microbiota may be one of the main reasons for this, although these metabolites have potential pharmacological activities. Liquiritigenin has various biological properties, such as anti-inflammatory, antioxidant, antiulcer, and antitumor effects [22,23]. In recent years, with in-depth research on natural medicines, many natural medicines have been found to have broad-spectrum antitumor effects. Liquiritigenin is a plant-derived, highly selective estrogen receptor β agonist. Many studies have found that liquiritigenin and its derivatives have positive effects on prostate cancer, breast cancer, ovarian cancer, oral cancer, glioblastoma, liver cancer, and other tumors. Broad-spectrum antitumor effects have attracted much attention [8,11,24,25,26,27]. However, many natural products have disadvantages, such as poor oral absorption and low bioavailability. Liquiritigenin, a flavonoid extracted from the Chinese herbal medicine *Glycyrrhiza uralensis*, is no exception. As a newly identified organ of the human body, the intestinal flora has attracted increasing attention from scientific researchers. The intestinal flora may be involved in the metabolism of the oral natural medicine liquiritigenin. Therefore, our attention is not limited to the prototype drug itself, as its metabolites may play a very important role in the biological effects of this drug. Through in vitro experiments, we determined the metabolism of liquiritigenin in the gut microbiota and liver microsomes and clarified the corresponding metabolic pathways, which has guiding significance for clinical translation.

The multiple biological effects of liquiritigenin after oral administration may be inseparable from the roles played by the gut microbiota and liver. Therefore, this study revealed the involvement of the gut microbiota in the liquiritigenin metabolism after using two systems (the gut microbiota and liver microsomes) and analyzed the five possible metabolites by mass spectrometry. Among them, liver microsomes metabolism produced two metabolites, M1 and M2, with molecular weights of 254 and 272, respectively. After comparing the extracted ion chromatograms of metabolites M1 and M2 with the standard samples, M1 and M2 were determined to be 7,4’-dihydroxyflavone and naringenin, respectively. This indicates that liquiritigenin is indeed metabolized in liver microsomes. R. Kupfer et al. found that liquiritigenin metabolism produces 7,4’-dihydroxyflavone through the in vitro metabolism of human liver microsomes of liquiritigenin [19]. We verified the metabolism of liquiritigenin to produce 7,4’-dihydroxyflavone by rat liver microsomes. R. Kupfer et al. found that no metabolite was detected in most plasma samples, and only the metabolite was occasionally detected in urine and feces, suggesting that the phase II metabolism of liquiritigenin may be related to it [19]. Y. K. Lee et al. demonstrated that isoliquiritigenin was metabolized into liquiritigenin, and liquiritigenin was widely metabolized into glucuronidated isoliquiritigenin and glucuronidated liquiritigenin by using the pharmacokinetics of isoliquiritigenin in rats [20]. Other studies have found many conjugates of liquiritigenin, such as glucuronide, sulfate, and glutathione, through in vivo metabolism [28,29,30]. It is well-known that flavonoids are absorbed in the intestine and undergo phase II metabolism in the liver to form glucuronized, sulfated, or/and methylated compounds [31,32,33]. However, in this study, only phase I metabolites 7,4’-dihydroxyflavone and naringenin were found due to liquiritigenin metabolism in liver microsomes in vitro, but phase II metabolites were not found. Therefore, the phase II metabolism of liquiritigenin in vivo needs further study in the future.

In this study, we also focused on characterizing the three metabolites of liquiritigenin generated after incubation with the gut microbiota by LC/MS-8060 and LC/MS^n^-IT-TOF analyses (M3, M4, and M5). M3 and M4 were determined to be phloretic acid and resorcinol, respectively, according to comparison with the standard samples.

We also found metabolite M5 in the intestinal flora system, and its [M-H]^-^ peak was found at 257.0822. M5 has two more hydrogens than liquiritigenin. According to the literature, dihydroflavonoids undergo ring cleavage at positions 1 and 2 under the actions of chalcone isomerase (CHI) and phloretin hydrolase (PHY) to produce metabolites by hydrogenation and reduction reactions [21]. It is therefore speculated that the structure of M5 may be dihydroisoflavone. The *m*/*z* of the secondary fragment of M5 was found at 151.0620, with a loss of 106 Da from the parent ion, which may be due to the loss of p-methylphenol. G. Yang et al. pointed out that flavonoid reductase specializes in catalyzing the hydrogenation of the C2-C3 double bond of flavonoids/flavonols a key step in the metabolism of these compounds [21]. Liquiritigenin produced M5 after intestinal flora metabolism, which again verifies the role of flavone reductase. Comparing the fragmentation information of M5 to the data from a literature report [34,35,36], M5 is likely to be davidigenin. Davidigenin belongs to the class of dihydrochalcone compounds and inhibits aldose reductase and improves diabetic complications [34]. T. Asano et al. used the human colon cancer cell line Caco-2 to detect the transepithelial flux of davidigenin and found that davidigenin has excellent absorption in this human intestinal epithelial cell line [37]. Davidigenin shows stronger and broader anticancer activity potential and dose-dependent antioxidative stress protection due to its similar chemical structure to the natural antitumor active ingredient isoliquiritigenin [38,39,40]. However, this hypothesis needs further verification. In conclusion, we confirmed that liquiritigenin can be metabolized in vitro by the intestinal flora and that flavonoid reductase in the intestinal flora can cleave liquiritigenin to produce metabolites.

There are certain unavoidable limitations of this study. First, we used intestinal contents and liver microsomes from a single species. Notably, there are differences in the composition and distribution of the intestinal flora among different species and individuals. The enzymes and other media produced by the liver may be different, which may lead to different results. Therefore, it is necessary to verify these findings in different species to expand the significance of this experiment. Second, it is necessary to explore the relationship between phase II metabolism and phase I metabolism of liquiritigenin and the effect of intestinal flora on liquiritigenin metabolism through in vivo experiments. Finally, the pharmacological effects of the metabolites produced by liquiritigenin deserve further study to reveal the internal mechanism of the broad-spectrum biological effects of this compound.

This study focused on the biotransformation of liquiritigenin by the intestinal microbiota through HPLC–MS/MS and LC/MS^n^-IT-TOF. Three metabolites were identified in the intestinal microbiota culture system, and two metabolites were identified in the liver microsomal metabolism system, which provided a clear metabolic pathway of liquiritigenin in the gut microbiota and liver microsomes. Gut microbiota metabolism and liver microsomal metabolism may provide a theoretical basis for the study of the pharmacologically active substances of flavonoids in vivo. With further research on the biological activities of gut microbiota metabolites, the pharmacodynamics and mechanisms of liquiritigenin and its metabolites are expected to be explored to guide future studies of the pharmacologically active substances of flavonoids in vivo.

## 4. Materials and Methods

### 4.1. Instruments and Reagents

Liquiritigenin (CAS: 578-96-9) was purchased from Shanghai Standard Technology Co., Ltd. (Shanghai, China). Naringenin (CAS: 480-41-1), 7,4’-dihydroxyflavone (CAS: 2196-14-7) and phloretic acid (CAS: 501-97-3) were purchased from Solarbio Life Sciences Co., Ltd. (Beijing, China). Resorcinol (CAS: 108-46-3) was purchased from Rhawn Chemical Technology Co., Ltd. (Ron Reagent) (Shanghai, China). The purity of the above compounds was greater than 98% by HPLC. HPLC-grade acetonitrile and acetic acid were purchased from Fisher Scientific (Fair Lawn, NJ, USA). Sprague–Dawley (SD) rat liver microsomes were purchased from RILD Research Institute for Liver Diseases Co., Ltd., Shanghai (cat number: WWJW). The structures of liquiritigenin and its metabolites from the intestinal flora and liver microsome systems were identified and analyzed by LC/MS-8060 and LC/MS^n^-IT-TOF systems from Shimadzu (Kyoto, Japan). An adjustable vortex mixer (VORTEX-GENIE2WH-681) was purchased from Scientific Industries, Bohemia, NY, USA. A small refrigerated high-speed centrifuge (Eppendorf Centrifuge 5424 R) was purchased from Eppendorf (Hamburg, Germany).

### 4.2. Animals

Three Sprague–Dawley (SD) rats (200–300 g, male) were provided by Vital River Laboratory Animal Technology Co., Ltd. (Beijing, China). These animals had free access to food and water and were housed on a 12 h light/dark cycle at 22–24 °C with 40–60% relative humidity. This study was conducted with the permission and guidance of the Experimental Animal Ethics Committee of the Chinese Academy of Medical Sciences and the Peking Union Medical College. All steps follow the “Organizational Guidelines and Ethics Guidelines of the Experimental Animal Ethics Committee”.

### 4.3. Determination of Liquiritigenin by LC/MS^n^-IT-TOF

To identify the metabolites of liquiritigenin, an LC/MS^n^-IT-TOF equipped with an ESI source was used. Analytes were separated using Luna C_18_-HST column (50 × 2 mm, 2.5 µm, Phenomenex, Torrance, CA, USA). The temperature of the column oven was 40 °C, and the flow rate was 0.4 mL/min. The mobile phase consisted of acetic acid and water (0.05:100, *v*/*v*) (mobile phase A) and acetonitrile (mobile phase B). The binary gradient elution method (A:B) was as follows: 2 min, 85:15; 7.00 min, 1:99; 9.00 min, 1:99; 9.01 min, 95:5; 12.00 min, stop. MS conditions: ionization mode, ESI source; analysis mode, negative ion mode; nebulizing gas flow rate, 1.5 L/min; CDL temperature, 200 °C; heating block temperature, 200 °C; detector voltage, 1.75 KV; collision energy, 50%; drying gas pressure, 115 kPa; mass spectrometry primary data acquisition range, *m*/*z* 100 to around 1000; and multilevel automatic data acquisition mode.

### 4.4. Determination of Liquiritigenin by HPLC–MS/MS

Liquiritigenin and its possible metabolites were analyzed with an LC–MS/MS 8060 system with an ESI ion source. Analytes were separated using a Luna C_18_-HST column (50 × 2 mm, 2.5 µm, Phenomenex, Torrance, CA, USA). The temperature of the column oven was 40 °C, and the flow rate was 0.4 mL/min. The mobile phase consisted of acetic acid and water (0.05:100, *v*/*v*) (mobile phase A) and acetonitrile (mobile phase B). The binary gradient elution method (A:B) was as follows: 2 min, 85:15; 7.00 min, 1:99; 9.00 min, 1:99; 9.01 min, 95:5; 12.00 min, stop. MRM mode was used for detection by the mass spectrometer, with mass transitions for liquiritigenin (negative MRM) of 255.15→119.10 (Q1 pre bias: 10.0 V, CE: 24.0 V, Q3 pre bias: 13.0 V, dwell time: 20 msec), 7,4’-dihydroxyflavone (negative MRM) of 253.15→116.90 (Q1 pre bias: 10.0 V, CE: 27.0 V, Q3 pre bias: 12.0 V, dwell time: 20 msec), naringenin (negative MRM) of 271.0→119.10 (Q1 pre bias: 22.0 V, CE: 25.0 V, Q3 pre bias: 21.0 V, dwell time: 20 msec), phloretic acid (negative MRM) of 165.30→93.10 (Q1 pre bias: 18.0 V, CE: 17.0 V, Q3 pre bias: 10.0 V, dwell time: 20 msec), and resorcinol (negative MRM) of 109.20. The mass spectrometer parameters were set as follows: nebulizer gas, 3.0 L/min; heating gas, 10 L/min; interface temperature, 300 °C; DL temperature, 250 °C; heat block temperature, 400 °C; drying gas, 10 L/min; interface voltage, −4.5 kV; and CID gas pressure, 270 kPa. All samples were maintained at 4 °C before injection.

### 4.5. In Vitro Incubation of Liquiritigenin with Liver Microsomes

The liver microsome incubation system consisted of the following: 5 μL of Sprague–Dawley rat liver microsomes (20 mg/mL), 2 μL of liquiritigenin (10 μg/mL, final concentration in the system was 1 µmol/mL), 20 μL of NADPH and 173 μL of Tris/HCl (0.05 mM, pH = 7.4) in a total volume of 200 μL. The cells were cultured in a shaking incubator at 37 °C and 800 rpm with oxygen. Incubation was terminated at 0, 15, 30, 60, 90, and 120 min, and 3 volumes of pure methanol were added to the incubation system to stop the reaction. After centrifugation at 13,400× *g* for 10 min in a refrigerated centrifuge at 4 °C, 100 µL of the supernatant was added to a chromatographic autosampler for analysis.

### 4.6. In Vitro Incubation of Liquiritigenin with the Gut Microbiota

The colon contents of three male Sprague–Dawley (SD) rats (200–300 g, fasted for 12 h before the experiment, purchased from Vital River Laboratory Animal Technology Co., Ltd. (Beijing, China)) were collected after sacrifice, and sterilized anaerobic medium (Solarbio Life Sciences Co., Ltd. (Beijing, China)) was added at a *m*/*v* ratio of 1:20 (g/mL), which was mixed evenly and purged with nitrogen after filtering. The mixture was preincubated at 37 °C for 60 min under anaerobic conditions. A methanol solution of liquiritigenin (2 mg/mL) was prepared, and 10 μL of this solution was added to a presterilized centrifuge tube (the final concentration of liquiritigenin in the system was 20 μg/mL), which was mixed with 990 μL of the preincubated mixture under anaerobic conditions. The drug was incubated with the gut microbiota at 37 °C for 0, 6, 12, and 24 h. In addition, a negative control containing heat-inactivated gut microbiota was incubated with liquiritigenin for the same length of time (24 h). After incubation, 3 volumes of pure methanol were added to the culture medium to stop the reaction and precipitate the protein. After centrifugation at 13,400× *g* for 10 min in a refrigerated centrifuge at 4 °C, 100 µL of the supernatant was added to a chromatographic autosampler for analysis.

### 4.7. Statistical Analysis

Data acquisition and processing were performed with Shimadzu LC–MS Solution (version 5.89, Kyoto, Japan). Two-tailed ANOVA and Student’s t test were used for statistical analysis with GraphPad Prism version 9 for macOS (GraphPad Software, San Diego, CA, USA). Data are expressed as the mean ± standard deviation (SD), and *p* values less than 0.05 were considered statistically significant.

## Figures and Tables

**Table 1:** Characteristics of the liquiritigenin metabolites from the gut microbiota system by LC/MS^n^-IT-TOF.

	Substance	Reaction	Theoretical Molecular Weight	Molecular Formula	Fragment Characteristics		
					MS^1^/[M-H]^-^	MS/MS	MS^3^
Gut microbiota system	Liquiritigenin	-	256.0736	C_15_H_12_O_4_	255.0678	135.0900 119.1453	91.0937
	M5	+2H	258.0892	C_15_H_14_O_4_	257.0822	151.0620 135.0957	

**Table 2:** The accurate mass measurements of molecular and fragment ions of liquiritigenin and its metabolites in the intestinal microflora metabolic system and liver microparticle incubation system.

	Substance	Experimental Molecular Weight	Theoretical Molecular Weight	Predicted Molecular Formula	Ion Mode	Diff (ppm)
	Liquiritigenin	255.0678	256.0736	C_15_H_12_O_4_	[M-H]^-^	5.93
Liver microsomes system	M1	255.0670	254.0579	C_15_H_10_O_4_	[M+H]^+^	7.14
	M2	273.0736	272.0685	C_15_H_12_O_5_	[M+H]^+^	7.90
Gut microbiota system	M3	165.0577	166.0630	C_9_H_10_O_3_	[M-H]^-^	0.11
	M4	111.0429	110.0368	C_6_H_6_O_2_	[M+H]^+^	10.51
	M5	257.0822	258.0892	C_15_H_14_O_4_	[M-H]^-^	1.03

## Data Availability

The data in this study are available in this article.

## Sample Availability

Samples of the compounds are available from the authors.
